# Glomus tumor in the stomach: A case report and review of the literature

**DOI:** 10.3892/ol.2014.1986

**Published:** 2014-03-21

**Authors:** KAI-BO CHEN, LI CHEN

**Affiliations:** Department of General Surgery, The Second Affiliated Hospital, College of Medicine, Zhejiang University, Hangzhou, Zhejiang 310000, P.R. China

**Keywords:** gastric glomus tumor, immunohistochemistry, diagnosis, treatment

## Abstract

This study reports a rare case of a 47-year-old female with a gastric glomus tumor who was admitted with epigastralgia. Endoscopic ultrasound revealed a protrusion on the posterior wall of the gastric antrum. Enhanced computed tomography confirmed the presence of a 10-mm mass. The tumor was resected, and immunohistochemistry revealed the tumor to be positive for smooth muscle actin and collagen type IV, and negative for synaptophysin, chromogranin A, laminin, S-100, cluster of differentiation (CD)34, CD31, CD99, cytokeratin (AE1/AE3), desmin and epithelial membrane antigen. The proliferation marker Ki-67 was positive in <5% of tumor cell nuclei. The clinical procedures with a review of the literature are reported.

## Introduction

Glomus tumors are benign lesions that originate from modified smooth muscle cells of the glomus body that help regulate arteriolar blood flow. These tumors are commonly observed in the dermis or subcutis, but are rarely located in the stomach, occuring in ~2% of all benign gastric tumors (1). Gastric glomus tumors are most commonly described as solitary, well-defined, submucosal lesions in the antrum, presenting with a variety of symptoms. Gastrointestinal bleeding with hematemesis/melena and epigastric discomfort are the most common initial symptoms and, in rare cases, may be life-threatening or lead to severe chronic anemia. Nausea and vomiting can also occur (2,3). Surgery is often performed promptly since malignancy cannot be excluded due to the rarity of this tumor (4). Gastric glomus tumors have a good prognosis due to low recurrence and rarity of malignant transformation. However, a long follow-up is required for further study. Patient provided written informed consent.

## Case report

A 47-year-old female was admitted to The Second Affiliated Hospital of Zhejiang University (Hangzhou, China) on March 20, 2013 due to intermittent epigastralgia for four months, which could be temporarily relieved by eating. The patient denied any associated weight loss, fevers, chills, nausea, vomiting or melena. Acid suppression therapy had been administered with only minimal relief. A physical examination revealed only mild tenderness in the epigastric area, and serum levels of tumor makers were all within normal limits. The patient’s father had died of gastric carcinoma.

**Figure 1:**
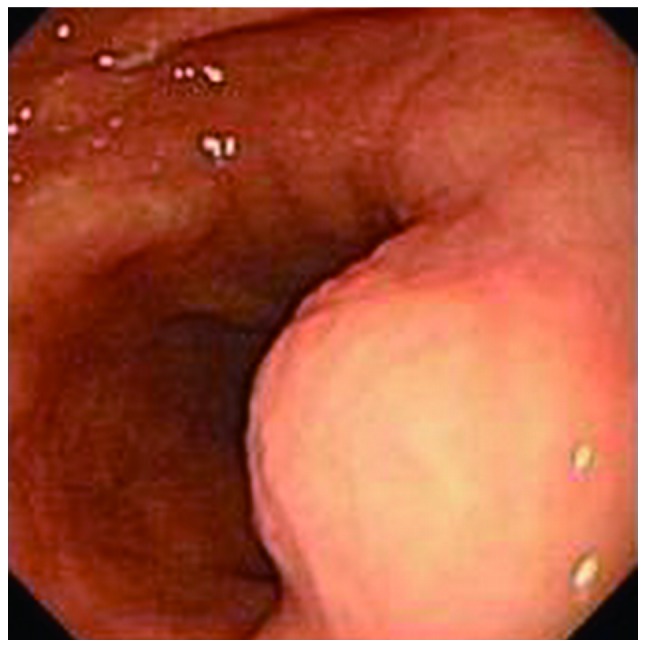
Endoscopy revealed a solid, elevated mass 20 mm in diameter.

**Figure 2:**
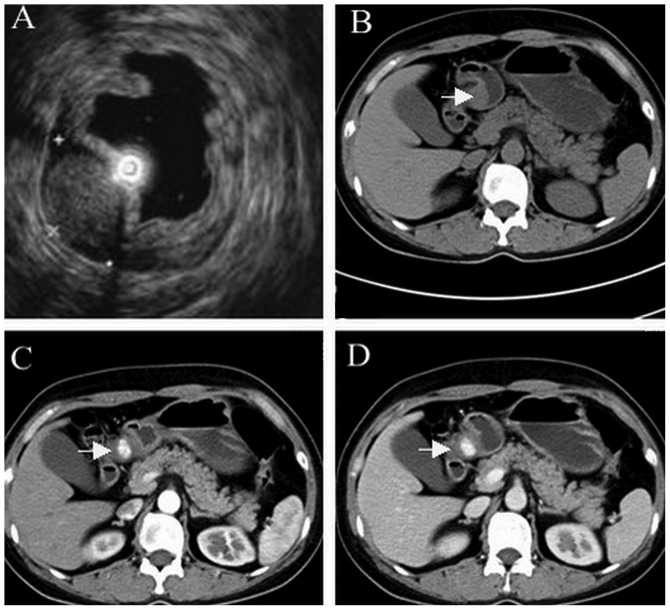
(A) EUS an ovoid, demarcated, heterogeneous, hyperechoic tumor 18.6×11.8 mm in size, originating from the fourth EUS layer (muscularis propria). Abdominal computed tomography revealed a well-demarcated, ovoid mass at the antrum (arrow) on (B) unenhanced, (C) arterial- and (D) delayed-phase scans. EUS, endoscopic ultrasound.

Endoscopic ultrasound (EUS) revealed a protrusion on the posterior wall of the gastric antrum (Figs. 1 and 2A). Computed tomography (CT) scan identified a mass on the antrum ~10 mm in diameter (Fig. 2B–D).

**Figure 3:**
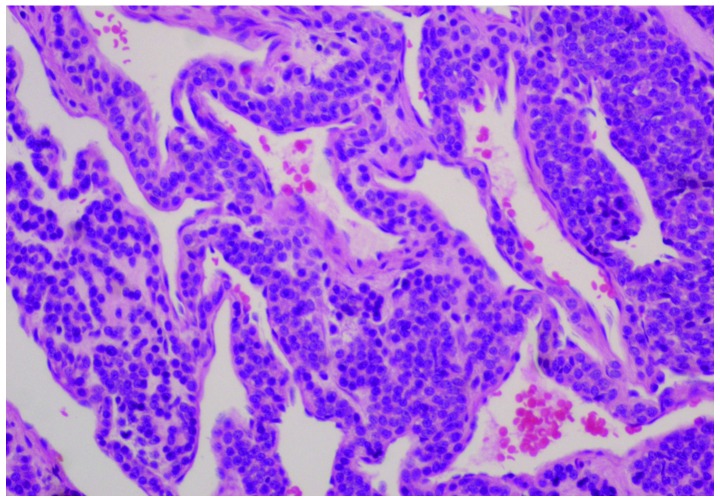
Microscopic examination revealed numerous dilated, thin-walled blood vessels, lined by a single layer of endothelial cells and surrounded by multilayer round glomus cells (hematoxylin and eosin staining; magnification, ×200).

**Figure 4:**
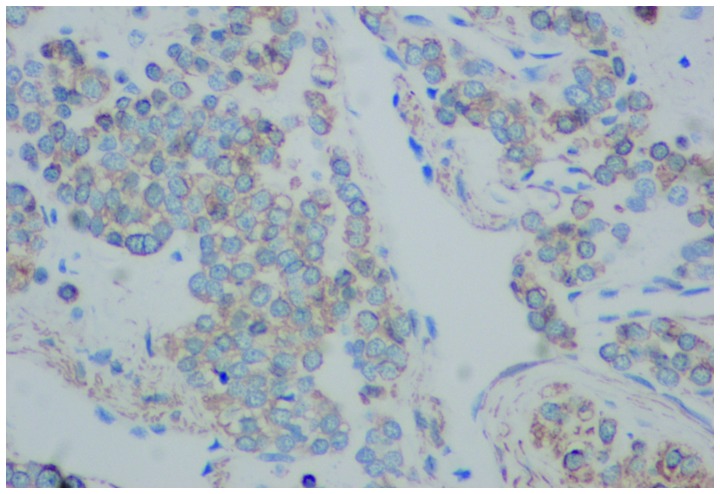
Tumor cells positive for smooth muscle actin, indicated by brown cytoplasmic stain (immunohistochemical staining; magnification, ×200).

**Figure 5:**
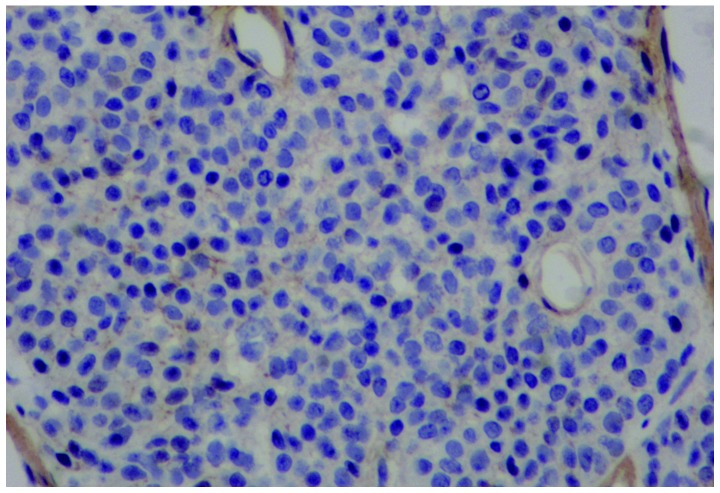
Tumor cells positive for collagen type IV (immunohistochemical staining; magnification, ×200).

Immunoperoxidase stains revealed positive staining for smooth muscle actin (SMA) and collagen type IV, while being negative for synaptophysin, chromogranin A, laminin, S-100, cluster of differentiation (CD)34, CD31, CD99, cytokeratin (AE1/AE3), desmin and epithelial membrane antigen. The proliferation marker Ki-67 was positive in <5% of tumor cell nuclei (Figs. 3–5).

A complete resection of the lesion was performed at The Second Affiliated Hospital of Zhejiang University. The patient was followed up for five months and recovered uneventfully without signs of relapse or gastrointestinal bleeding.

## Discussion

Glomus tumors are rare in the stomach, and were first reported by De Busscher in 1948 as benign lesions (5). Gastric glomus tumors are now defined as mesenchymal tumors with potential malignant behavior. Malignant glomus tumors of the stomach with various organ metastases have been reported (6,7). Criteria for identifying malignant potential in gastric glomus tumors remain to be established (8).

Pre-operative diagnosis of gastric glomus tumors is challenging and requires a multi-faculty medical approach. On unenhanced CT, they manifest as well-circumscribed submucosal masses with homogeneous density and may contain tiny flecks of calcification. Following the administration of contrast medium, these tumors demonstrate strong enhancement on arterial-phase scans and persistent enhancement on portal venous-phase scans (2). By contrast, the density of gastrointestinal stromal tumors is lower and these do not exhibit prolonged enhancement in the delayed phase (9). EUS features of gastric glomus tumors are heterogeneous, hypoechoic or hyperechoic, and hypervascular masses with internal hyperechoic spots and few tubular structures, mostly located on the fourth echolayer (10,11). CT and EUS are useful in the early identification of gastric glomus tumors, particularly in terms of assessing tumor blood supply (12). On magnetic resonance images, gastric glomus tumors are marginally hypointense on T1-weighted images, slightly hyperintense on T2-weighted images, and hypervascular. In addition, gastric glomus tumors exhibit persistent enhancement following gadopentetate dimeglumine administration (13). Immunohistochemistry (IHC) is the preferred diagnostic tool, by which distinctive small, uniform and round tumor cells surrounding capillaries can be found, which are strongly positive for SMA, vimentin, calponin, collagen type IV and laminin (14).

Fine needle aspiration (FNA) can distinguish glomus tumors from more aggressive gastric tumors rapidly and pre-operatively, avoiding extensive surgical resection, particularly in larger tumors (8,15,16). However, FNA can also incorrectly diagnose glomus tumors as leiomyomas or well-differentiated neuroendocrine tumors (2). Recently, Mohanty *et al* reported a case of gastric glomus tumor diagnosed by EUS-guided FNA and cell block IHC prior to endoscopic submucosal resection (ESMR) (17).

Complete surgical excision is the optimal treatment for a single lesion (18), although subtotal gastrectomy has been proposed for tumors suspected of malignancy (1). To minimize surgical trauma and the inflammatory response, the benign nature and small median size [varying between 2 and 3 cm (19)] of glomus tumors allows them to be removed by laparoscopic wedge resection (9,20–22), or endoscopic submucosal enucleation in select cases (22,23) where the lesion is not close to the pylorus, porta hepatis and along the lesser curvature (24).

In summary, gastric glomus tumors are rare solitary submucosal tumors for which pre-operative diagnosis is challenging. Imaging findings assist significantly in making differential diagnoses. FNA with IHC may also be a promising method of diagnosis, helping the surgeon to plan an ESMR rather than more radical surgery. Exact diagnosis relies on histopathological examinations. Local resection by open or laparoscopic surgery is usually the most efficient therapy, although endoscopic submucosal enucleation may be another effective treatment.
